# Renal dysplasia with single system ectopic ureter: Diagnosis using magnetic resonance urography and management with laparoscopic nephroureterectomy in pediatric age

**DOI:** 10.4103/0970-1591.57916

**Published:** 2009

**Authors:** Milind Joshi, Sandesh Parelkar, Heemanshi Shah

**Affiliations:** Department of Paediatric Surgery, Seth G.S.M.C. and K.E.M. Hospital, Mumbai, India

**Keywords:** Laparoscopic nephroureterectomy, magnetic resonance urography, single system ureteral ectopia

## Abstract

Single system ureteral ectopia and associated congenital dysplastic kidney is surgically curable etiology of incontinence with other wise normal pattern of voiding in female child. We share our experience of eight cases in last one year and its management with laparoscopic nephroureterectomy at a tertiary care hospital in India which is one of the largest series in such a short duration of this rare anomaly.

**Materials and Methods:**

Patients presented with clinical features of continence with otherwise normal pattern of voiding were clinically examined and investigated by ultrasound (USG), nuclear renal scan, magnetic resonance urography (MRU). Laparoscopic nephroureterectomy was done in all the eight cases and renal dysplasia was confirmed on histological examination.

**Results:**

All the patients were females in the age group of five months to five years. USG detected the renal dysplasia in three out of eight cases; however, it could not detect the course of the ectopic ureter in any of the cases. MRU picked up the dysplastic moieties and their location as well as functional status and also depicted the course of the ectopic ureter opening into the vaginal wall in all the eight cases. Laparoscopic nephroureterectomy was done in all the cases and patients were cured off their symptoms.

**Conclusion:**

Single system ectopic ureter associated with congenital renal dysplasia is exceedingly rare. MRU is definitely the better investigation for the diagnosis of this condition as compared to the conventional radiological investigations. Laparoscopic nephroureterectomy is a very good procedure for the management of these cases.

## INTRODUCTION

A single system ectopic ureter may be defined as a ureter that opens caudal to the bladder trigone in a nonduplicated system. They present predominantly in female children with typical symptom of incontinence with otherwise normal pattern of voiding.[1] Compared with ectopic ureter draining the duplicated kidney, the diagnosis is delayed because the associated small dysplastic kidney and nonfunctioning renal tissue is not picked up by conventional radiological investigations. We discuss our experience of such eight cases in last one year where confirmation of the diagnosis was done using MRU. Laparoscopic nephroureterectomy was done in all of them. This is the largest series of this rare anomaly diagnosed with MRU and managed laparoscopically in such a short duration reported so far.

## MATERIALS AND METHODS

Patients presenting with complaints of the continence with incontinence were clinically evaluated to rule out any associated urogenital, neurological abnormalities, spina bifida. All the patients underwent ultrasound as an initial screening investigation. Nuclear renal scans, MRU was done using heavily T2- weighted images, contrast enhanced T weighted MR sequences and maximum intensity projection (MIP) after proper hydration in 1.5T MR scanner imaging. Very young and uncooperative patients were given oral sedation.

Post contrast images in T1 sequences were obtained and results were analyzed.

Laparoscopic nephroureterectomy was done under general anesthesia with endotracheal intubation. The procedure was carried out in supine position with 15° elevation of the operating table on the affected side and head low position. Urinary bladder was catheterized and three ports of 5 mm each were used. Umbilical port for camera, flank port on the opposite side of the affected kidney and suprapubic port as the working ports were used. Carbon dioxide was used for creating pneumoperitoneum and pressure was 8–10 mm of Hg at a rate of 1 l/min. Transperitoneal laparoscopy was done. Ureter was first identified where it crosses the iliac vessels at the pelvic brim and the dysplastic kidney was traced upwards. The dilated ureter was punctured if needed and aspirated after incising the peritoneum over it. The vessels of the dysplastic kidney were coagulated with bipolar diathermy. The lower end of the ureter was traced as low as possible up to their ectopic openings up to the vaginal wall, ligated and cut. The specimen of the kidney and the ureter was removed from the suprapubic port site by extending the incision to 1.0 to 1.5 cm .if required. Port sites were closed with absorbable sutures. The specimen was removed from suprapubic port by extending the incision to 1–1.5 cm if required and sent for histological confirmation of the renal dysplasia.

## RESULTS

Total eight children presented with such complaints in last one year at our center and all of them were female with age range of five month to five years. None of them had any neurological abnormalities or any other complaints. One patient had anterior perineal anus and was deflating well, others had normal anal opening.

USG was indicative of small dysplastic kidney in three patients (*n* = 3) with associated hydroureter but the entire course of the ureter couldn't be seen in them. Renal scan showed no function on the affected side with normal function on the opposite side.

MRU detected dysplastic renal tissue in all the eight patients (*n* = 8) in the renal fossa on one side with normal functioning kidney on the other side. The complete course of the ureter of this kidney, its caliber, its termination into ectopic site in all cases was seen.

Two patients had ectopic ureter with dysplastic kidney on left side and the rest six had right side affected. Laparoscopic nephroureterectomy was done in all the eight patients. Average duration of the procedure was 60 min (range 40 min to 90 min). The size of the dysplastic kidneys ranged from 1.5 × 1.0 × 1.2 cm to 3.0 × 2.0 × 1.5 cm. and all were present in the renal fossa. All the patients had associated hydroureter of variable size. None of the patients had any intraoperative or postoperative complication. All were allowed orally on the same day of surgery and were discharged after 48 h. All of them are continent and doing well.

## DISCUSSION

The ectopic ureter with congenital renal dysplasia classically presents in female patients as continence with in continence.[1] It is one of the surgically curable causes of incontinence, hence requires proper diagnosis and surgery. Ureteral ectopia can be unilateral or bilateral, with duplicated ureter or single ureter and its associated malformations.[2] In most cases, ectopic ureter is associated with ureteral complete duplication. Single ureteral ectopia and associated congenital renal dysplasia are rare malformations. According to Chinese birth defect monitoring center, the incidence is 29 per million.[3]

According to the hypothesis by Stephens, the further the ureteral orifice is located from the trigone of the bladder, the more severe is the ipsilateral renal dysplasia. Once the ectopic ureteral orifice is found in the vestibule or vagina, renal dysplasia always concurs and vice versa.[2] Single ureteral ectopia encompasses a spectrum of the malformations involving the bladder trigone, ureter and kidney.[4] The clinical presentations are variable and diagnostic and therapeutic problems are common. It is important to localize the associated dysplastic kidney and to differentiate single ureteral ectopia with solitary kidney from single ureteral ectopia with congenital renal dysplasia.[5]

IVU has no role in the diagnosis of single system ureteral ectopia with renal dysplasia as there is no excretion of the contrast on the affected side. USG as an initial screening method along with color Doppler is used to rule out duplex system and to find out presence of dysplastic kidney and its localization either at ectopic site or in the renal fossa and for vascularity of kidney. It can also detect associated hydroureter if the system is obstructed. However, it is highly observer dependent and the dysplastic renal tissue may not be easy to see as was clearly evident in our cases where in only three out of eight cases, dysplastic kidney was detected, giving it a sensitivity of 40% only.

Renal scans are not very useful in diagnosing such cases as the dysplastic renal tissue has hardly any function and such systems are usually obstructed rather than refluxing system. Gangopadyaya *et al*. have reported DMSA renal scans and IVU as diagnostic modalities in their series of ectopic ureter.[6] However, we feel that both these investigations are little useful for the diagnosis of this condition.

CT urography with contrast with 3-D reconstruction of the images has also been used as an investigation in some of the published studies.[2,7] However, it has the disadvantage of very high radiation exposure and risk of allergic reaction to the contrast agents and hence we did not use this investigation in our cases.

**Figure 1:**
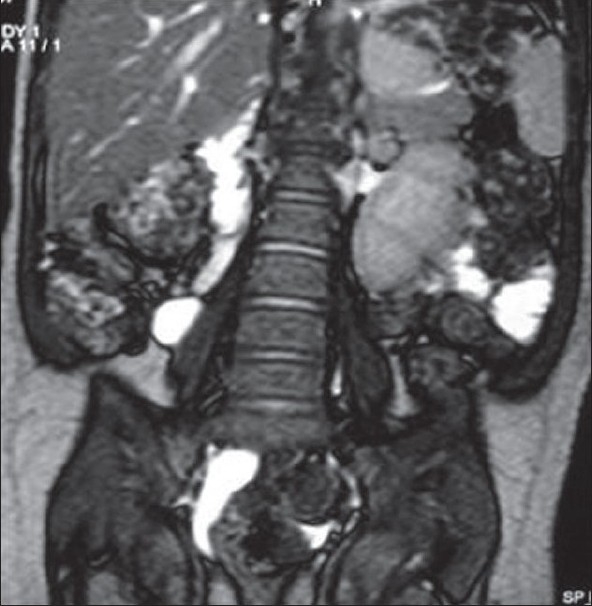
MRU showing dysplastic right kidney with dilated right ectopic ureter going beyond urinary bladder T2 W IMAGE

**Figure 2:**
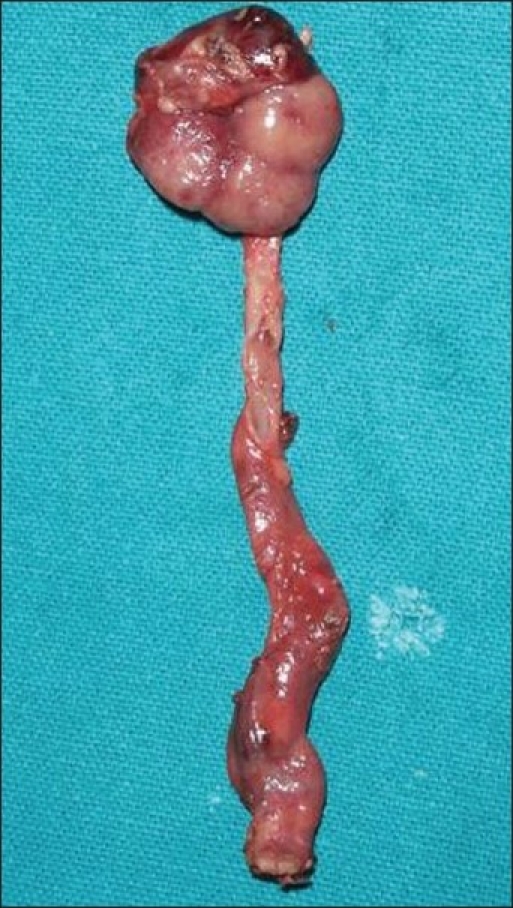
Excised specimen of nephroureterectomy

MRU is an excellent modality of investigation in such cases.[8] The dysplastic renal tissue is specifically picked up on T1W and T2W images and course of the ectopic ureter could also be seen very well in all the cases. The T2W images picked up the dysplastic kidney and the hydroureter as hyper intense images [Figure 1] and T1W images [Figure 2] were hypo intense for them.[9,10] Gadolinium contrast images gave excellent picture of functioning renal tissue and ureter and could be differentiated from nonfunctioning tissue.[11] 3-D reconstruction with maximum intensity projection (MIP) of the images gave very clear anatomical and functional picture of normal as well as pathological system like none of the conventional imaging. It also had the advantage of no radiation exposure and as a single modality of investigation could detect the ectopic ureter with dysplastic kidney.[12]

In our cases, we found it to be 100% sensitive and specific as it could preoperatively detect the position of the dysplastic kidney and the ectopic opening of the draining ureter. Using MRU, we could diagnose the pathology without any undue delay which was the common problem mentioned in the series by Borer *et al*.[4] The other advantage of this preoperative localization was that during the laparoscopic extirpation of the kidney, we did not have to dig up for its localization which could have been otherwise quite cumbersome because of the small size of the dysplastic tissue.

Cystogenitoscopy and ascending urography can be done if the ectopic ureteric orifice is seen and cannulated. The evidences on Cystogenitoscopy for the presence of ectopic ureter are noticed as bulging of the anterior vaginal wall, presence of the hemitrigone and absent ureteric opening at the normal site. We did not perform cystoscopy in our patients for the diagnosis of this condition as MRU gave sufficient information; however, Cystogenitoscopy can be helpful to rule out bladder neck pathology as a cause of urinary incontinence and with above described findings does help in making the diagnosis of the ectopic ureter especially where the facility for MRU is not available.

Once the diagnosis is confirmed and opposite kidney is normally functioning, the procedure of choice is to remove the dysplastic kidney and the ureter. Conventionally, open surgery is done for the same. However, laparoscopic nephroureterectomy was done as an alternative to the open surgery as reported by Koyle *et al*.[13] We also used it in all our eight cases.

None of the patients had any intraoperative or postoperative complications. Average duration of the procedure was 1 h. Postoperative analgesia was required only as a single dose of paracetamol in the dose of 10 mg/kg intravenous route. Patients were allowed orally after 4 h of the procedure and were discharged after 48 h. We reviewed the reported series and case reports of laparoscopic nephroureterectomy.

**Figure 3:**
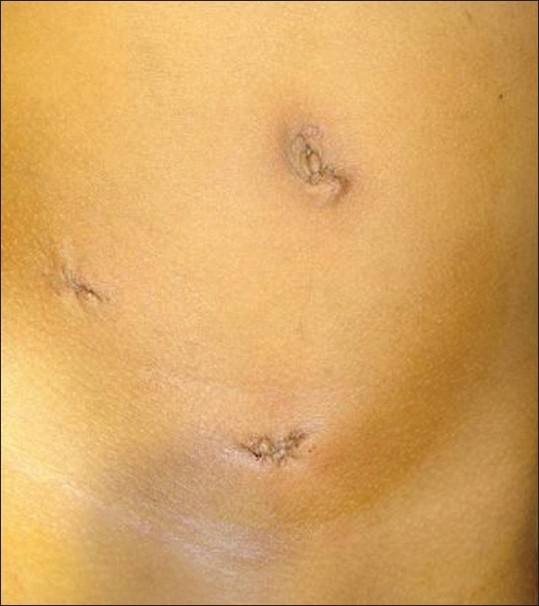
Sutured port sites of left laparoscopic nephroureterectomy

We have used three ports methods [Figure 3] as compared to four ports used in the case report by Moores *et al*.[14] The procedure time and postoperative recovery is comparable to the series reported by Kurokawa *et al*.[15] and Kim *et al*.[16]

## CONCLUSION

Our experience of the single system ectopic ureter and associated congenital dysplastic kidney found MRU as an excellent investigation for preoperative diagnosis, exact location of the dysplastic kidney, course of the ectopic ureter and hence no need to dig up for searching the dysplastic kidney during surgery. Laparoscopic nephroureterectomy is a very good method for the treatment of these cases and we strongly recommend this procedure for such patients.
